# Disentangling the contribution of multiple land covers to fire‐mediated carbon emissions in Amazonia during the 2010 drought

**DOI:** 10.1002/2014GB005008

**Published:** 2015-10-22

**Authors:** Liana Oighenstein Anderson, Luiz E. O. C. Aragão, Manuel Gloor, Egídio Arai, Marcos Adami, Sassan S. Saatchi, Yadvinder Malhi, Yosio E. Shimabukuro, Jos Barlow, Erika Berenguer, Valdete Duarte

**Affiliations:** ^1^ National Center for Monitoring and Early Warning of Natural DisastersSão José dos CamposBrazil; ^2^ Environmental Change InstituteUniversity of OxfordOxfordUK; ^3^ Remote Sensing DivisionNational Institute for Space ResearchSão José dos CamposBrazil; ^4^ College of Life and Environmental SciencesUniversity of ExeterExeterUK; ^5^ School of GeographyUniversity of LeedsLeedsUK; ^6^ Jet Propulsion LaboratoryCalifornia Institute of TechnologyPasadenaCaliforniaUSA; ^7^ Institute of EnvironmentUniversity of CaliforniaLos AngelesCaliforniaUSA; ^8^ Lancaster Environment CentreLancaster UniversityLancasterUK

**Keywords:** Amazonia, fire, drought, carbon emission, biomass

## Abstract

In less than 15 years, the Amazon region experienced three major droughts. Links between droughts and fires have been demonstrated for the 1997/1998, 2005, and 2010 droughts. In 2010, emissions of 510 ± 120 Tg C were associated to fire alone in Amazonia. Existing approaches have, however, not yet disentangled the proportional contribution of multiple land cover sources to this total. We develop a novel integration of multisensor and multitemporal satellite‐derived data on land cover, active fires, and burned area and an empirical model of fire‐induced biomass loss to quantify the extent of burned areas and resulting biomass loss for multiple land covers in Mato Grosso (MT) state, southern Amazonia—the 2010 drought most impacted region. We show that 10.77% (96,855 km^2^) of MT burned. We estimated a gross carbon emission of 56.21 ± 22.5 Tg C from direct combustion of biomass, with an additional 29.4 ± 10 Tg C committed to be emitted in the following years due to dead wood decay. It is estimated that old‐growth forest fires in the whole Brazilian Legal Amazon (BLA) have contributed to 14.81 Tg of C (11.75 Tg C to 17.87 Tg C) emissions to the atmosphere during the 2010 fire season, with an affected area of 27,555 km^2^. Total C loss from the 2010 fires in MT state and old‐growth forest fires in the BLA represent, respectively, 77% (47% to 107%) and 86% (68.2% to 103%) of Brazil's National Plan on Climate Change annual target for Amazonia C emission reductions from deforestation.

## Key Points

- The 10.77% (96,855 km2) of MT burned during the 2010 dry season
- Mato Grosso gross c emission in 2010 was 56.21 ± 22.5 Tg C
- Old‐growth forest fire emission in 2010 in the Brazilian Amazon was 14.81 Tg C

## 1 Introduction

Severe climatic events in Amazonia, such as droughts or floods, occur with a frequency of about 10 years [*Marengo*, 2009]. However, in recent years, the Amazon region experienced three major droughts (1997/1998, 2005, and 2010), suggesting a tendency for increased dry events with longer dry seasons in the south and south‐west flanks of the basin, that may be exacerbate in the next decades [*Marengo et al.*, 2011]. The increased intensification and frequency of these drought events, in addition to the observed recent floods [*Espinoza et al.*, 2013; *Satyamurty et al.*, 2013], seem to be consistent with previous modeling results predicting the increase in the frequency of extreme events throughout the 21st century [*Malhi et al.*, 2008]. However, the underlying physical processes driving these observed changes in climate are not fully understood. It has been argued that these processes could be driven by (i) the warming of surface waters of the tropical Pacific and Atlantic Oceans, inducing more frequent El Niño‐ or Atlantic Multidecadal Oscillation‐driven droughts, respectively [*Cai et al.*, 2014; *Cox et al.*, 2008], (ii) the intensification of atmospheric Walker circulation in cooling the sea surface in eastern Pacific [*McGregor et al.*, 2014], or (iii) the organized deep convection atmospheric process [*Tan et al.*, 2015]. These processes are not necessarily mutually exclusive.

There is a close link between droughts and fires in the Amazon [*Barbosa and Fearnside*, 1999; *Alencar et al.*, 2005; *Aragão et al.*, 2007; *Chen et al.*, 2011]. A multitude of socioeconomic‐ecological impacts associated to the effects of fires in Amazonia have been reported, such as forest biomass reduction due to long‐term increases in large‐tree mortality [*Barlow et al.*, 2003], change in tree species composition [*Barlow and Peres*, 2008], and reduction of agricultural production and increase in rural property damages [*de Mendonça et al.*, 2004]. Fires are not exclusively a significant source of carbon to the atmosphere [*van der Werf et al.*, 2010], but the large‐scale transport of atmospheric aerosol during these fires can also have significant negative impacts on human health [*Smith et al.*, 2014], airport operation, and other socioeconomic activities [*Anderson et al.*, 2011].

The net carbon sink in the Brazilian Amazon during nondrought years can be reverted during drought years, with forest fires contributing to approximately 25% of the emissions [*Aragão et al.*, 2014]. During dry years, fires associated with deforestation, pasture cleaning, and agricultural lands often leak to the surrounding forests and properties [*Aragão et al.*, 2007]. Moreover, it has been shown that 59% of the areas with decreased deforestation trend between 2000 and 2007 in the Brazilian Amazon were experiencing increased fire trend [*Aragão and Shimabukuro*, 2010].

Despite the indication that fires are becoming an increasingly important component of the Amazonian carbon budget, even with the remarkable reduction of Brazilian Legal Amazon (BLA) deforestation rates to 4848 km^2^ yr^−1^ in 2014, the literature lacks information on the contribution of different land covers affected by fire to the total aboveground C emissions of this region. Currently, most attempts to calculate C emissions from the BLA only account for the contribution of fire emissions related to the clear‐cut of undisturbed forests and in few cases clear‐cut of regenerating vegetation (secondary forests) growing in previously deforested lands [*Aguiar et al.*, 2012]. Neglecting fluxes from other land covers is likely to underestimate the actual impact of fires upon the Amazonian net C budget, with critical implications for defining effective targets for C emission reduction in Brazil.

The Decree No. 7.390/2010, which regulates the Brazilian National Plan on Climate Change, projects that C emissions from all sectors by 2020 in Brazil will be 0.87 Pg C, from which 0.25 Pg C corresponds to land cover conversion in the Amazon. This same decree establishes a target of reducing the projected values from all sectors to values between 0.53 Pg C and 0.55 Pg C. Based on atmospheric greenhouse gas profiles measured over the Amazon using aircraft, *Gatti et al.* [2014] estimated that during the 2011 anomalously wet year, fires were responsible for 0.30 ± 0.10 Pg C, a value similar to the projected C emission reduction proposed for all sectors by 2020 (~0.33 Pg C). The same study quantified that during the extreme dry year of 2010, fires were responsible for emitting 0.51 ± 0.12 Pg C to the atmosphere, a value close to the targeted emission proposed by the Brazilian government for the year 2020.

To better understand the role of fires on C emissions and effectively support and drive the development of mitigation strategies, including fire prevention and management, it is necessary to break down emissions into natural forest and savannah fires, land management fires, and deforestation‐related fires [*Balch*, 2014]. Moreover, it is important to quantify the role of the different fire types, as only wildfires in denser vegetation or fires associated with deforestation represent a long‐term net source of CO_2_ to the atmosphere. CO_2_ emissions from fires used to clean and manage pastures and agricultural fields and from grasslands such as some Brazilian cerrado formations may be balanced out by carbon uptake during the vegetation regrowth in the following growing season.

Analyses to date on carbon emissions from fires do not cover the entire range of land cover types and are either based only on few high‐resolution satellite data [*Alencar et al.*, 2006; *Morton et al.*, 2011, 2013; *Oliveras et al.*, 2014] or are based on large spatial resolution data (0.5°). Uncertainties associated to higher‐resolution approaches are likely to be lower than low‐resolution approaches, if the aim is to understand variation in land cover types. However, high‐resolution approaches may be subject to increased uncertainty if the results need to be extrapolated to larger areas [*van der Werf et al.*, 2010].

In this study, we provide a comprehensive assessment of the spatial extent and patterns of burned areas and associated aboveground carbon emissions from fires for different land cover types for one of the regions most affected by the 2010 drought—Mato Grosso state, in southern Brazilian Amazon [*Marengo et al.*, 2011]. Our study uses (i) a wall‐to‐wall land cover map encompassing land use information from 1980s to 2010; (ii) a high‐quality assessment of cumulative burned areas from June to October 2010; and (iii) a statistical model of fire‐induced biomass loss, which uses as an input state of art estimates of aboveground biomass [*Saatchi et al.*, 2011]. We specifically aim to answer four research questions: (1) What was the extent of the burned area in 2010? (2) Which land cover types were most impacted by fires? (3) How much carbon was lost from the aboveground biomass in the multiple land cover types due to fires in 2010? (4) What are the main uncertainties of these estimates?

## 2 Materials and Methods

We addressed our research questions by (1) generating a wall‐to‐wall burned area map for Mato Grosso state derived from a linear spectral mixing model applied to Moderate Resolution Imaging Spectroradiometer (MODIS) surface reflectance images; (2) combining the cumulative burned area map with a land cover map for 2010 for extraction of areal extent of burned area by land cover; and (3) producing a per‐pixel carbon loss estimate by combining information from maps of aboveground biomass (AGB), land cover, and burned areas with an empirical relationship between biomass before and after fire. To estimate the contribution of each land cover type to the total carbon emission, we converted the pixel‐based biomass loss for each land cover type into carbon by using emission factors available in the literature.

### 2.1 Study Area

Mato Grosso state has an area of approximately 900,000 km^2^ and is located in the southern part of the Brazilian Legal Amazon, encompassing the cerrado and Amazon biomes in its southern and northern boundaries, respectively. The diversity of vegetation types found in this area is a result of natural environmental variability and the spatiotemporal variations in climate, including the length of the dry season and rainfall patterns. Human activities are also important determinants of land cover patterns in this region. Mato Grosso state has been one of the Amazonian states with higher deforestation rates and fire detections. However, since 2006, deforestation rates in the region have decreased, despite the increase in agricultural production (soybean and cattle) [*Macedo et al.*, 2012].

### 2.2 Data

#### 2.2.1 Land Cover Map

The area of different land covers within Mato Grosso state was derived from the Panamazônia II project at a scale of 1:500,000 [*Shimabukuro et al.*, 2010; *Martini et al.*, 2012]. The methodology uses data from Landsat Multispectral Scanner System of 1980s, Landsat Thematic Mapper of 1990s, Landsat Enhanced Thematic Mapper Plus of 2000s, and the 2009 and 2010 data from Terra Moderate Resolution Imaging Spectroradiometer (MODIS) based on a multiresolution and multitemporal methodology [*Shimabukuro et al.*, 2010].

**Figure 1:**
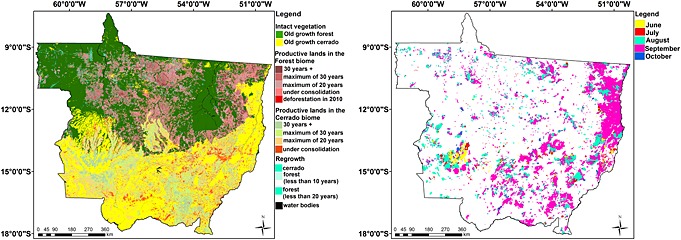
Land cover and burned areas in Mato Grosso state with 250 m spatial resolution. (a) Mato Grosso land cover map for 2010, derived from Panamazônia II project. (b) Cumulative burn scars map, from June to mid‐October 2010.

The 2010 map of Mato Grosso (Figure 1a) presents four groups of classes:

1. Intact vegetation: old‐growth tropical forest and old‐growth cerrado.
2. Productive lands in forest and cerrado biomes: stratified by the number of years it has been under production (areas under use for more than 11 years) and under consolidation (areas under use for 10 years or less).
3. Regrowth: forest or cerrado regrowth.
4. Deforestation in 2010: deforestation in old‐growth tropical forest and deforestation of forest regrowth (detailed methods and map accuracy assessment are available in Text S1 and Table S1 in the supporting information).

#### 2.2.2 Biomass Map

For the aboveground biomass, we used the 1 km resolution AGB map and error map developed by *Saatchi et al.* [2011]. This map was generated based on a combination of ground and remote sensing data for the year 2000. The AGB and associated AGB error maps were used to estimate the average biomass for each land cover type (detailed information available in Text S2 and Table S2). The AGB and error maps were also used to estimate the biomass of the postfire remaining forests using a field‐based empirical relationship between live AGB before fire and remaining live AGB after fire (more details are provided in sections below). The resulting values from the application of this relationship to the biomass map were subsequently used for calculating the per‐pixel fire‐related AGB loss for each land cover type.

#### 2.2.3 Active Fires

We used monthly active fire data, at 1 km spatial resolution, from MODIS‐Terra sensor product MCD14ML collection 5 for the period between January 2001 and December 2010 to support the burn scar mapping and to extrapolate the burned area measurements and carbon emissions from forests to the whole Brazilian Legal Amazon (BLA). During ideal observational conditions (near‐nadir and reduced smoke), flaming fires with 100 m^2^ size on the ground can be detected by MODIS 1 km resolution products [*Giglio*, 2010]. The product may underestimate the occurrence of fires in situations including cloudy conditions, the start and end of fire between satellite overpasses, fires occurring under the forest canopy, and too small or too cool fires to be detected at 1 km^2^ MODIS pixel resolution. Overestimation of fire pixels in the MODIS product may be associated with targets with contrasting temperatures (e.g., forest‐bare soil boundary in a warm day) and sandy soils or exposed rocks, which can have high temperature in warm days [*Schroeder et al.*, 2008]. For this analysis, we only considered high‐confidence fire pixels (above 80% confidence level, according to the MODIS product ancillary information) and fire pixels located inside mapped burned areas.

A preliminary analysis of active fire pixels in Mato Grosso for the period from 2001 to 2010 showed that for this period, 80% to 95% of all high‐confidence fire detections occur between June and October. Thus, the burn scar detection (section below) aimed to cover these months of high fire occurrence (fire season) (Figure S1 in the supporting information).

#### 2.2.4 Burn Scar Mapping

We mapped burn scars using daily surface reflectance products MOD09GA and MOD09GQ as well as 8 day surface reflectance products, MO09Q1 and MOD09A1, collection 5 from the MODIS data set. The dates of the images were selected based on the latest day of the month with nadir view and cloud‐free images for the study area. The 2010 daily images were acquired on 28 June, 25 July, 24 August, 25 September, and 13 October. The 2010 8 day mosaic image dates were acquired on 28 June, 24 July, 23 August, 12 September, and 13 October. This analysis included the red (band 1, 620–670 nm) and near‐infrared (NIR; band 2, 841–876 nm) reflectance bands from MOD09GQ (daily) and MOD09Q1 (8 day) products and shortwave infrared (SWIR; band 6, 1628–1652 nm) from MOD09GA (daily) and MOD09A1 (8 day) products. The minimum detected burned area is assumed to be approximately 25 ha (4 pixels of 250 m × 250 m). The burned area maps were generated following the methods developed by *Anderson et al.* [2005], *Shimabukuro et al.* [2009], and *Lima et al.* [2012].

In general terms, the methodology for detecting burned areas is based on the linear spectral mixing model applied on RED, NIR, and SWIR MODIS spectral bands of the daily and 8 day composite MODIS products. The shade fraction image is the main source of information for mapping burnt areas. A segmentation procedure was applied to the shade fraction images using a minimum area threshold of 4 pixels with a digital number similarity up to eighth. Subsequently, we performed an unsupervised classification using ISOSEG [*Bins et al.*, 1993] and a postclassification image edition [*Shimabukuro et al.*, 1998]. The postclassification image edition was carried out by a skilled human interpreter using the natural color composites of the corresponding images for comparison. This task minimizes the omission and commission errors normally produced by any classification algorithm [*Almeida‐Filho and Shimabukuro*, 2002]. The regions mapped as burned areas in one date were used as a mask in the following dates. The mask is temporally consistent due to the high quality of the subpixel geolocation accuracy of the MODIS land products [*Wolfe et al.*, 2002]. Consequently, no spatial information on areas that burnt more than once were recorded, and the final map represents the cumulative burnt areas from June to mid‐October 2010 (Figure 1b).

We performed an accuracy assessment of the burn scars mapped using as references images from Landsat 5 and Landsat 7 (Anderson et al., under review). A total of 208 Landsat scenes, available at http://glovis.usgs.gov/, covering a period between June and October 2010, were used for validation (Table S4 in the supporting information). The overall accuracy of burned forests were 99.20% (97.67% to 99.48%) and for nonforest burned areas were 96.93% (93.76% to 98.92%) (Table S4 in the supporting information).

#### 2.2.5 Biomass Loss and Committed Carbon Emissions

**Figure 2:**
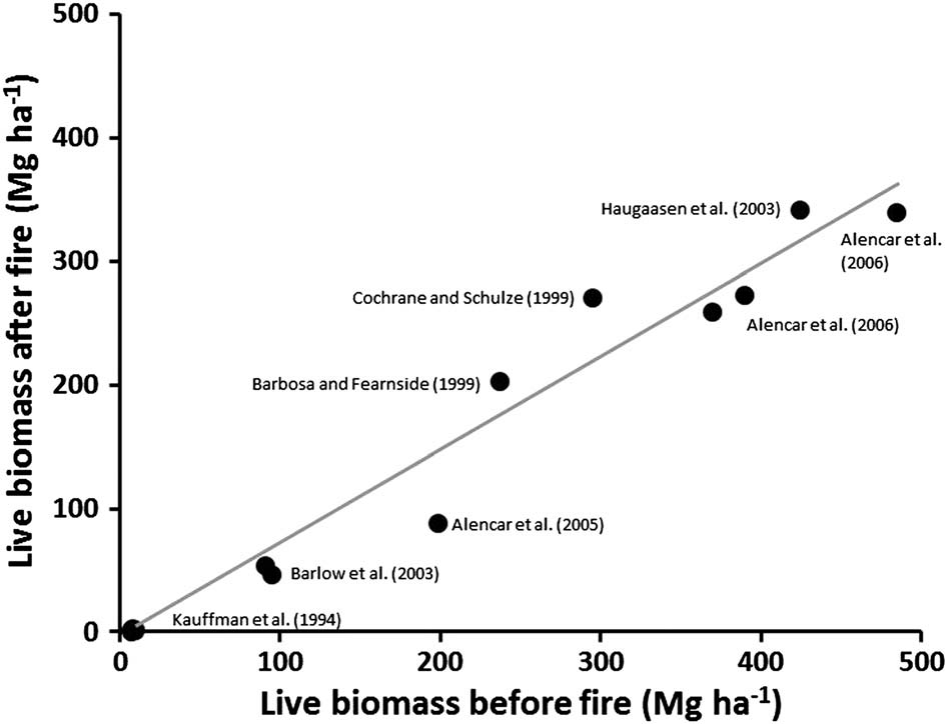
Relationship between initial biomass and remaining biomass after fire events. Plot data compiled from the literature have burned only once with measurements taken within 1 year of the fire event. The complete references and supporting information for each measurement are presented in the Table S5.

To quantify AGB loss due to fire events, we first hypothesized that the remaining live biomass in an area affected by fire is strongly correlated with the initial biomass before fire. This hypothesis is based on the fact that as the biomass increases, microclimate inside the canopy tends to become wetter and cooler reducing the intensity and suitability for fire spread [*Brando et al.*, 2012]. Therefore, we expect biomass to be an integrator of complex interactions between climate and fire within the canopy that are directly related to the intensity and consequent impact of fires on biomass. To test this hypothesis we quantified the relationship between initial live biomass before fire and live biomass remaining after fire by compiling, from the literature, independent biomass information observed at forest census plots before and after a natural fire occurrence (Table S5). We found a highly significant linear relationship between biomass before and after fire (Figure 2), (*R* ^2^ = 0.95, *P* < 0.01; Text S5 and Table S5). This equation was subsequently used to calculate the pixel‐based biomass loss for each land cover class (equation (1)).

(1) $$B_{f}=\;0.7084*B_{i}\;\text{.}$$

*B_f_* is the remaining aboveground live biomass (Mg ha^−1^) after fire, and *B_i_* is the initial aboveground live biomass (Mg ha^−1^) of the plot.

This linear relationship between AGB before and after fire derived from the literature is subject to errors in both axes, *x* and *y*, and it may vary from study to study. Therefore, the slope and its uncertainty was estimated using a linear regression which takes into account both *x* and *y* errors [*York et al.*, 2004]. The uncertainty (standard deviation) of the slope was ±0.034 (Mg Mg^−1^). All values compiled for establishing the equation were derived from measurements of AGB within 1 year after the fire occurrence. This method only accounts for the short‐term (1 year) carbon loss. Long‐term biomass losses (>3 years) may be larger due to the delayed mortality of large trees reported for 3 years after a fire event [*Barlow et al.*, 2003].

### 2.3 Data Analysis

#### 2.3.1 Burned Area and Biomass Loss

For analyzing the extent of burned area and the associated biomass loss, for each burned grid cell (250 m) we extracted information on the aboveground live biomass, the aboveground biomass error, and the land cover class. The mean value per land cover class is presented in Table S3. We subsequently applied equation (1) for each pixel containing AGB values to estimate the after fire AGB and its uncertainty (details below). Finally, we calculated the AGB loss and associated uncertainty for each pixel within each land cover type as part of the calculations depicted in equation (2), below (Table 2).

#### 2.3.2 Gross and Committed Emissions

Carbon emission from the biomass affected by fire was calculated following two alternative pathways: (1) AGB could be immediately released to the atmosphere through direct combustion of the organic material or (2) fire could kill the vegetation, moving its AGB to the decomposing pool, where it would be slowly released through the decomposition process. Our analysis treated these two pathways separately. We first produced an estimate of the annual gross emission, which refers to the C that was immediately released to the atmosphere by direct combustion plus the C emitted from decomposition in the first year after fire. We also estimated the committed emission, which refers to the total amount of the remaining C that is eventually released to the atmosphere by decomposition of dead organic matter. We assumed that 48% of dry biomass is carbon [*Ward and Hardy*, 1991].

To estimate the 2010 gross emission, three pathways were considered, depending on the land cover and land use type:

1. *Cerrado grasslands and productive lands (pastures and agriculture):* all biomass loss calculated using equation (1) is combusted and thus instantly released to the atmosphere.
2. *Old‐growth forests and forest regrowth from areas cleared in 2010:* we assumed that 50% of the biomass removed is immediately combusted (gross emission in 2010) and the rest entered the decomposing pool (committed emissions) [*van der Werf et al.*, 2009]. Combustion completeness of large stems (>10 cm diameter breast high (DBH)) is lower than 5% in slash and burn experiments [*Carvalho et al.*, 1998; *Araújo et al.*, 1999].
3. *Old-growth forest and forest regrowth*: we assumed that there is no direct combustion of the AGB affected by fires in these areas. All the biomass loss is related to mortality and therefore directly transferred to the decomposing pool. Emissions for these two land cover types were calculated based on an annual decay rate of 0.17 yr^−1^ [*Chambers et al.*, 2000]. Only C losses for the first year of decay were attributed to gross emission in 2010, and the remaining material was assumed to stay in the decomposing pool. The direct emissions from the burned litterfall, wood debris, and soils were not accounted for as these represent a relatively small portion of total biomass in forest systems [*Berenguer et al.*, 2014].

For estimating the total gross C emission in 2010, we summed the first year carbon losses from decomposition (old‐growth and secondary forest areas) and the directly combusted carbon (cerrado, cerrado regrowth, and productive areas). Committed emissions were estimated for old‐growth and secondary forests by subtracting the total carbon loss by the total gross emission in 2010 (first year of emissions from decomposition). In this paper we only assess the contribution of the AGB to the total carbon emissions.

In summary, our model of gross emission follows:

(2) $$F\;=\lambda_{\left(\text{ldcover}\right)}\cdot \;B_{i\left(x,y\right)}\cdot \;\left(1‐\alpha_{\left(x,y\right)}\right)\;dA_{\left(x,y\right)}\text{,}$$

where

1. *F* (ton yr^−1^) is the carbon gross emission (immediate flux to the atmosphere);
2. *λ*_(ldcover)_ is the decay or release constant (yr^−1^) specific to each land cover type (ha). Identification of land cover type has an accuracy of 80% (i.e., in 80% of the cases the land classification is correct, with a confidence interval from 74% to 87%);
3. *B*_*i*(*x*,*y*)_ is the preburn biomass density (Mg ha^−1^) for the pixel at location (*x*,*y*) provided by *Saatchi et al.* [2011];
4. *α* is the slope of the equation (1) (*α* = 0.7084 ± 0.034); and
5. *dA*_(*x*,*y*)_ is the burned area (in ha) at pixel with location (*x*,*y*), with an accuracy of 99.2% (confidence interval of 97.67% to 99.48%) for forests and an accuracy of 96.93% (confidence interval of 93.76% to 98.92%) for the nonforest classes.

#### 2.3.3 Scaling‐Up Burnt Area and Biomass Loss Using Active Fire Data

After calculating the fire‐affected area and the committed carbon emission, we quantified the total number of active fire pixels for each land cover type affected by fire. These data were used to perform a weighted least squares (WLS) regression between the old‐growth forest area affected by fire (dependent variable) and the cumulative active fire pixels (explanatory variable) from June to October 2010 within the burn scars. This relationship was used to quantify the impact of fires on Amazonian old‐growth forests outside the boundaries of Mato Grosso state. The WLS was used as the standard deviation of the random errors in the data were not constant across all levels of the variables [*Carroll and Ruppert*, 1988]. Weights of the regression were generated based on a preliminary ordinary least squares (OLS) fit [*Bloomfield*, 2014] (Figure S2). The WLS analysis encompasses the uncertainties for burned area and fire pixels, which are grouped into classes, and higher weights are given to the group of samples with residuals closer to zero [*National Institute of Standards and Technology*/*Semiconductor Manufacturing Technology Consortium*, 2015]. This analysis demonstrated the potential of using active fire information to estimate the burned area and thus biomass loss due to fires over a larger area. This is especially relevant as active fire data have a higher temporal resolution and require less computational effort and processing time, facilitating initial assessment of fire impact over large regions.

After statistically formalize the relationship between old‐growth forest burned area as a function of the cumulative active fire pixels, we estimated the gross C emissions for this vegetation type for the whole Brazilian Legal Amazon (BLA). To separate old‐growth forests from other vegetation types we used the 2010 forest mask derived from the Monitoring the Brazilian Amazon Gross Deforestation Project ‐ PRODES [*Instituto Nacional de Pesquisas Espaciais (INPE)*, 2015]. Accumulated fire pixels from June to 13 October 2010 that superposed the forested area were filtered according to the product's quality flags (>80% confidence level). Uncertainty in the extrapolation analysis was carried out by varying the biomass loss equation's slope by its minimum and maximum values (0.6744 to 0.7424) according to *York et al.*'s [2004] method.

### 2.4 Uncertainty Assessment

In addition to the AGB map error provided by *Saatchi et al.* [2011], we compared *Saatchi et al.*'s [2011] AGB map with other available biomass data sets in the literature: *Saatchi et al.* [2007] and *Baccini et al.* [2012], presented in Text S3 and Table S3. We have also performed an analysis to evaluate the uncertainty of our gross emission estimate by applying a Monte Carlo approach with 100 repetitions for each component of equation (2). This was achieved by varying randomly those parameters, which are approximately normally distributed (the slope of before and after fire biomass relation, and prefire, also called initial biomass). For categorical variables (discrete data: land cover type and burned versus unburned (i.e., 0 or 1)) we used a threshold criteria based on the map accuracy. These variables were tested using a uniformly distributed random number generator.

## 3 Results

### 3.1 Extent of Burned Areas During the 2010 Drought

A total area of 96,855 km^2^ (93,990 km^2^ to 101,209 km^2^), corresponding to 10.4% to 11.2% of the Mato Grosso state area, burned at least once from June to mid‐October 2010. These fires affected 31% to 33% of the total pristine vegetation remaining in Mato Grosso. Fires spread over 60,507 km^2^ (58,589 km^2^ to 63,629 km^2^) of the cerrado biome (26.5% to 28.8% of the cerrado area) and 12,975 km^2^ (12,776 km^2^ to 13,011 km^2^) of old‐growth forests (4.41% to 4.49% of the forest areas). In addition, 22,158 km^2^ (21,456 km^2^ to 23,301 km^2^) of productive lands, corresponding to 47.3% to 51.3% of all pastures and agricultural land area, and 22% to 23.8% (962 km^2^ to 1045 km^2^) of secondary forests and cerrado regrowth also burnt (Table 1).

**Table 1:** Total Count of High‐Confidence Active Fire Pixels in Burned Areas and Cumulative Burnt Area, in km^2^ (Lower Limit; Upper Limit), Per Land Cover Type in 2010^a^

			Burned Area in 2010 (km^2^)						
	High‐Confidence Fire Pixel Counts (June to 13 October)	Area in 2010 (km^2^)	June	July	August	September	October	Total Burnt Area (km^2^) (Lower Limits; Upper Limits)	% of the Class Burnt (Lower Limits; Upper Limits)
Intact vegetation									
Old‐growth forest	2,326	289,358	152	191	1,704	10,362	566	12,975 (12,776; 13,011)	4.48 (4.41; 4.49)
Old‐growth cerrado	6,467	220,928	5,191	2,502	10,969	40,578	1,266	60,507 (58,589; 63,629)	27.39 (26.51; 28.8)
Productive lands in the forest biome									
Permanent productive for 30 years +	118	8,710	63	80	104	346	25	619 (599; 650)	7.11 (6.87; 7.46)
Permanent productive for maximum of 30 years	171	31,760	36	40	262	667	29	1,033 (1,000; 1,086)	3.25 (3.14; 3.41)
Permanent productive for maximum of 20 years	686	58,184	66	119	867	2,291	118	3,460 (3,350; 3,638)	5.95 (5.75; 6.25)
Under consolidation (productive for 10 years or less)	1,638	57,093	134	211	1,537	3,601	490	5,974 (5,784; 6,282)	10.46 (10.13; 11.0)
Deforestation in 2010	1	83	0	0	0	2.68	0.57	3.2 (3.0; 3.3)	3.93 (3.61; 3.97)
Productive lands in the cerrado biome									
Permanent productive for 30 years +	183	30,192	539	407	593	1,061	31	2,630 (2,546; 2,765)	8.71 (8.43; 9.15)
Permanent productive for maximum of 30 years	268	67,678	331	165	305	1,686	75	2,563 (2,481; 2,695)	3.79 (3.66; 3.98)
Permanent productive for maximum of 20 years	489	87,251	317	189	586	3,383	76	4,551 (4,406; 4,785)	5.22 (5.04; 5.48)
Under consolidation (productive for 10 years or less)	186	29,878	76	52	64	1,077	57	1,325 (1,282; 1,393)	4.44 (4.29; 4.66)
Regrowth									
Cerrado	30	1,777	7	4	20	161	3	196 (189; 206)	11.04 (10.63; 11.59)
Forest regrowth (less than 20 years)	1	50	0	0	0.23	2.48	0.25	3.1 (3.0; 3.2)	6.27 (6.0; 6.4)
Forest regrowth (less than 10 years)	128	14,178	5	10	202	455	123	795 (769; 836)	5.61 (5.42; 5.85)
Deforestation in 2010 on less than 10 years regrowth	32	2,524	7	11	24	160	18	221 (213; 232)	8.74 (8.43; 9.19)
Total	12,724	899,645	6,924	3,982	17,237	65,835	2,878	96,855 (93,990; 101,209)	10.77 (10.44; 11.24)

a The burned area limits (km^2^) were determined based on the map accuracy assessment (ESM Table 4).

**Figure 3:**
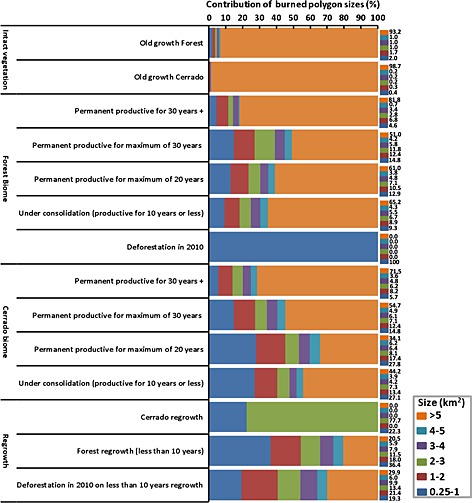
Contribution of burned areas by size per land cover type.

Large patches of burned areas (>5 km^2^) dominate the landscape in most land cover types, contributing to the majority of areas affected by fires. They corresponded to 93% and 99% of burned areas in intact forests and cerrado, respectively. The majority of the burned areas in the productive lands are larger than 3 km^2^. In contrast, the majority of burned areas in new forest clearings in 2010 as well as cerrado and forest regrowth areas were smaller than 2 km^2^ (Figure 3).

In total, more than 12,700 high‐confidence active fire occurrences were detected by MODIS active fire product from June to mid‐October 2010. Active fire pixels are effective for representing larger burned areas greater than 0.5 km^2^, which represent 24% of the total area burned, approximately 101,446 polygons. From the total number of high‐confidence active fire pixel occurrences, 50% were detected in the old‐growth cerrado, 18% were detected in old‐growth forests, 29% were detected over productive lands, and 0.5% were detected on forest and cerrado regrowth areas. September was the month with the highest number of active fire detections, corresponding to 70% of the total detections during the period analyzed.

### 3.2 Gross and Committed Emissions Due to Fires in 2010

The amount of AGB C lost from multiple land cover types as consequence of fires from June to mid‐October 2010 was estimated to be 85.3 ± 33.2 Tg (1 Tg = 10^12^ g). This corresponds to a gross emission in 2010 of 56.1 ± 22.5 Tg C and a committed carbon emission of 29.4 ± 10.0 Tg C (Table 2). The total carbon loss from intact vegetation (old‐growth forest and old‐growth cerrado) in Mato Grosso state was estimated to be 66.4 ± 26.2 Tg C, approximately 78% of the total carbon loss. Productive lands contributed with 9.68 ± 6.26 Tg, approximately 19% of the total C loss, which was accounted as gross or immediate emissions, and secondary vegetation contributed with a sum of 1.27 ± 0.78 Tg (2.5%). Fires in old‐growth forests correspond to approximately 38% of the total carbon loss, a value higher than the combined C losses from productive lands and secondary vegetation together (22.13%). Sixty percent of the emissions correspond to gross emissions, while the remaining 40% will be slowly released to the atmosphere through the dead tree decomposition process.

**Table 2:** Biomass of Affected Area, Biomass Loss, Gross, and Committed C Emissions Due to Fires Per Land Cover Type During the Dry Season in 2010^a^

	Biomass (Mg ha^−1^) of the Affected Areas	Biomass Loss Due to Fires (Mg ha^−1^)	Gross C Emission in 2010 (Tg)	Committed C Emission (Tg)	Total Carbon Loss (Tg)	% of Carbon Loss
‐	Mean (±Error)	Mean (±Error)	(±Total Error)	(±Total Error)	(±Total Error)	‐
Intact vegetation						
Old‐growth forest	176.3 (±34.63)	51.63 (±19.6)	5.05 (±1.92)	27.2 (±10.4)	32.3 (±12.28)	37.87
Old‐growth cerrado	40.23 (±33.43)	11.71(±4.79)	34.1 (±13.9)	‐	34.1 (±13.94)	39.98
Productive lands in the forest biome						
Permanent productive for 30 years +	53.69 (±33.27)	15.65 (±6.40)	0.46 (±0.19)	‐	0.46 (±0.19)	0.54
Permanent productive for maximum of 30 years	68.42 (±33.67)	19.95 (±8.16)	0.99 (±0.40)	‐	0.99 (±0.40)	1.16
Permanent productive for maximum of 20 years	105 (±34.62)	30.61 (±12.5)	5.13 (±2.10)	‐	5.13 (±2.09)	6.01
Under consolidation (productive for 10 years or less)	105 (±34.76)	17.29 (±7.07)	5.02 (±2.05)	‐	5.02 (±2.053)	5.89
Deforestation in 2010	208.9 (±33.24)	60.91 (±23.0)	4.78 × 10^−3^ (±1.81 × 10^−4^)	3.01 × 10^−2^ (±2.71 × 10^−2^)	9.56 × 10^−3^ (±3.61 × 10^−3^)	0.01
Productive lands in the cerrado biome						
Permanent productive for 30 years +	27.17 (±32.09)	7.92 (±3.24)	1.00 (±0.41)	‐	1.00 (±0.41)	1.17
Permanent productive for maximum of 30 years	33.5 (±32.39)	9.76 (±3.99)	1.22 (±0.49)	‐	1.22 (±0.49)	1.43
Permanent productive for maximum of 20 years	31.14 (±32.22)	9.08 (±3.71)	1.99 (±0.81)	‐	1.99 (±0.81)	2.33
Under consolidation (productive for 10 years or less)	31.14 (±32.61)	9.29 (±3.80)	9.75 × 10^−2^ (±1.22 × 10^−2^)	‐	9.75 × 10^−2^ (±0.21)	0.11
Regrowth						
Cerrado	37.51 (±32.55)	9.42 (±3.01)	9.21 × 10^−2^ (±3.76 × 10^−2^)	‐	9.21 × 10^−2^ (±3.76 × 10^−2^)	0.11
Forest regrowth (less than 20 years)	208.1 (±34.11)	43.38 (±16.4)	1.03 × 10^−3^ (±3.87 × 10^−4^)	5.53 × 10^−3^ (±2.09 × 10^−3^)	6.56 × 10^−3^ (±2.47 × 10^−3^)	0.01
Forest regrowth (less than 10 years)	148.8 (±34.82)	49.74 (±18.9)	0.30 (±0.11)	1.64 (±0.62)	1.95 (±0.74)	2.29
Deforestation in 2010 on less than 10 years regrowth	131.2 (±34.76)	85.27 (±0.0)	0.45 (±0.0)	0.45 (±0.0)	0.91 (±0.0)	1.07
Total	‐	‐	56.1 (±22.5)	29.4 (±10.0)	85.3 (±33.2)	100

a The error associated with the biomass of affected areas was derived from the AGB error map from *Saatchi et al.* [2011]. The errors associated with biomass loss, carbon loss, and emissions were derived from the Monte Carlo simulations.

### 3.3 Scaling‐Up Biomass Loss to the Brazilian Legal Amazon

**Figure 4:**
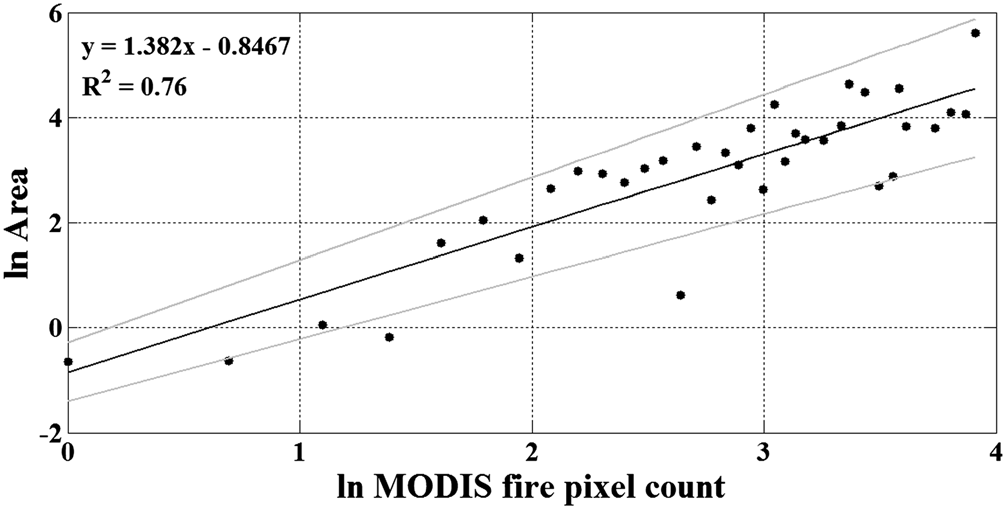
Weighted least squares regression (WLS) analysis between primary forest burned area (ha) and fire pixels (count) for Mato Grosso state. The upper and lower lines denote 95% confidence interval.

Our analysis indicates that old‐growth forest burnt areas are significantly related with the number of active fire pixels occurring within the burned scars (*P* < 0.001; Figure 4). We used this finding to extend the Mato Grosso analysis over the entire Brazilian Amazon. The total number of high‐confidence fire pixels from MODIS product MCD14ML for the Brazilian Legal Amazon (BLA) from June to 13 October 2010 was 49,885, with 13,667 detected in old‐growth forests. Using the relationship between fire pixel and burned area for old‐growth forests (Figure 4), approximately 14,580 km^2^ of old‐growth forests burned outside Mato Grosso state. Therefore, for the whole BLA the 2010 drought‐related fires have impacted an area of approximately 27,555 km^2^ of old‐growth forests. This area is approximately 4 times higher than the deforestation for the same year [*INPE*, 2015].

**Figure 5:**
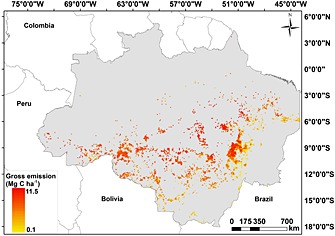
Carbon gross emissions from primary forests due to fires during the dry season in 2010.

By applying the same procedure for calculating committed and gross C emissions as described in the Methods section, we estimate that the gross carbon emission in 2010 due to fires in old‐growth forests for the whole BLA was 9.76 Tg C (8.62 Tg C to 10.9 Tg C) (Figure 5) and the total carbon loss is estimated to be 62.4 Tg C (55.1 Tg to 69.7 Tg).

## 4 Discussion

Our analysis provided the understanding of the impact of fires on multiple land cover types, which are normally not comprehensively accounted for, using moderate resolution data (250 m), in the attempts to estimate fire‐associated carbon emissions from Amazonia. By partitioning the impacts of these fires on the aboveground carbon for multiple land covers we provided strategic information for decision makers to tackle fire the problem in Amazonia. Based on our results, in this section we answer our four scientific questions.

### 4.1 What Was the Extent of the Burned Areas in 2010?

Based on the burned area map (Figure 1), we estimated that an area of about 97,000 km^2^ was burned in 2010 in the state of Mato Grosso alone. Fire impact may be higher if small fires are accounted for. In this study we have not considered burned areas smaller than 25 ha. None of the current estimates using MODIS data are able to capture small fires [*van der Werf et al.*, 2009; *Morton et al.*, 2011], which can be common in Amazonia [*Cardozo et al.*, 2014]. Second, our data reflect cumulative burned area. The map accuracy of this study burned areas was overall high for forests and nonforest areas (Table S4). In comparison, the MOD45 burned area product [*Roy et al.*, 2008] for the same period detected approximately 69,000 km^2^, about 30% less than our estimate. Moreover, due to the methods used in the MOD45 product generation, burn scars that persist for more than 1 month may be detected as a burned area in the following month (first author's observation). These discrepancies in burned area estimates by different methods emphasize the challenges for generating a fully automated burned area product for large areas, and the comparison among different data sets and methods are still part of active research in the scientific community [*Giglio et al.*, 2006; *Jain*, 2007; *Chuvieco et al.*, 2008; *Chang and Song*, 2009].

### 4.2 Which Land Cover Types Were Most Impacted by Fires?

Our results highlighted the growing importance of fires during years with abnormally low rainfall in intact forests and cerrado. During the 2010 dry season in Mato Grosso, about 12,776 km^2^ to 13,011 km^2^ of burned areas corresponded to forests and 58,588 km^2^ to 63,629 km^2^ to cerrado, totaling 73,482 km^2^ (71,365 km^2^ to 76,640 km^2^) of burned intact vegetation. On the other hand, productive lands contributed with 22,158 km^2^ (21,455 km^2^ to 23,301 km^2^), an area 3 times smaller than the pristine vegetation impacted by fires.

In the other two extreme droughts in the Amazon, intact forests and cerrado also burned. Approximately 11,394 km^2^ to 13,928 km^2^ of old‐growth forests and 23,971 km^2^ of cerrado burnt in Roraima state during the 1997/1998 El Niño event [*Barbosa and Fearnside*, 1999]. During the 2005 drought, 2800 km^2^ of old‐growth forests and 3700 km^2^ of productive lands burnt in Acre [*Shimabukuro et al.*, 2009]. *Morton et al.* [2013] mapped understory fires for the southern Amazon, including the BLA, northern Bolivia, and Southern Peru, and found that years with a large number of burned areas were 2005, 2007, and 2010, with approximately 14,400 km^2^; 25,600 km^2^; and 18,500 km^2^ burned, respectively.

### 4.3 How Much Carbon Was Lost From the Aboveground Biomass in the Multiple Land Cover Types Due to Fires in 2010?

In this study we estimated that 56.1 ± 22.5 Tg of carbon were released to the atmosphere due to fires during June to October 2010, in Mato Grosso state alone. When using the biomass map from *Saatchi et al.* [2007] and *Baccini et al.* [2012] instead of *Saatchi et al.* [2011], our gross emission estimates change to 40.7 Tg C and 89.8 Tg C, respectively (Table S3). While *Saatchi et al.*'s [2007] data set estimates are within the uncertainties, when considering all variables in the Monte Carlo simulation, the result based on *Baccini et al.*'s [2012] data set is marginally in the upper boundaries of the emission calculated for *Saatchi et al.* [2011].

For the following discussion it is convenient to partition carbon emissions into “rapidly reversible” (RR) emissions and the “long‐term reversible” (LTR) carbon emissions. RR emissions refer to emission from grass‐like vegetation (pastures, crops, and cerrado), where during a fire event, the fuel biomass lost on combustion varies from 49% to 91%, depending on the equation used for calculating the biomass loss [*Balch et al.*, 2011], and the biomass is recovered by regrowth within 1 year [*Oliveras et al.*, 2013]. Studies in the Brazilian cerrado have reported that the total biomass consumed by fires varies from 97 to 100% in cerrado grasslands and 72% to 84% in denser cerrado types [*Oliveras et al.*, 2013]. From our results, we estimated that 50.3 ± 20.5 Tg of C are RR emissions, released to the atmosphere between June and October in Mato Grosso state from grasslands and productive lands.

The LTR carbon emission refers to forests and forest regrowth. From our results, we estimated that LTR emissions of 5.81 ± 2.04 Tg C were released to the atmosphere from June to October 2010 in Mato Grosso state (gross emissions). The LTR‐committed C emission is estimated to be 29.4 ± 11.0 Tg released to the atmosphere through the decomposition of remaining dead biomass. It has been estimated that 16% of forests burn quite often, especially in the states of Mato Grosso and Pará [*Morton et al.*, 2013]. Fire incidence and recurrence in pristine forests in the Amazon can lead to a drastic and cumulative change in forest structure and composition [*Barlow et al.*, 2003; *Balch et al.*, 2011]. Therefore, the recovery through time in terms of both carbon and biodiversity in burned forest areas can be considered effectively irreversible.

The carbon loss from old‐growth forest fires in Mato Grosso (MT) (32.3 ± 12.2 Tg C) is higher than the total emission from Roraima during the El Niño 1997/1998 and is higher than the estimates of forest fires for the entire Brazilian Amazon in a non‐El Niño–Southern Oscillation phase (5.8 to 7.5 Tg C) [*Alencar et al.*, 2006]. The total carbon loss from the 2010 fires in Mato Grosso state (85.3 ± 33.2 Tg C) is 1.2 times higher than the yearly net deforestation emissions from 2001 to 2005 (66.6 Tg C) [*Aguiar et al.*, 2012] in this state—the period with the highest registered rates of deforestation, and higher than the 2008–2010 average Brazilian Legal Amazon net deforestation emissions (83.6 ± 28.6 Tg C) [*Song et al.*, 2015]. Moreover, by summing the carbon loss from the natural vegetation (old‐growth forest and cerrado) in Mato Grosso (219 ± 86.5 Tg CO_2_), it corresponds to 41% of the emissions (higher boundary) estimated for the Brazilian Amazonia during the El Niño phase in 1997/1998 (70 to 540 Tg CO_2_) [*Alencar et al.*, 2006], suggesting that the 2010 fire emissions during this major drought potentially exceed previous drought emissions.

### 4.4 What Are the Main Uncertainties of These Estimates?

In relation to the gross emissions estimated in this study, it is expect that possible overestimation may be due to postfire leftover debris, such as residual uncombusted fuel and incompletely combusted ash in the cerrado and productive lands. This overestimation is likely small though in our estimates as most of the fires occurred in the mid‐to‐end of the dry season, when the vegetation is dry and combustion is nearly complete.

Two sources of underestimates for quantifying carbon loss are identified in this study. First, underestimation could be associated with the linear relationship between biomass before and after fire. It is expected that when areas with low AGB burns, a comparable high proportion of the AGB is lost (e.g., cerrado grasslands). Conversely, as the AGB before fire increases, the proportional biomass loss decreases. This is coherent with the expectations that as biomass increases, microclimate inside the canopy tends to become wetter and cooler reducing the intensity and suitability for fire spread.

It is expected that cerrado and grassland biomass losses due to fires can vary from 72% to 97% [*Kauffman et al.*, 1994] depending on the vegetation and fire characteristics. Considering these thresholds, the carbon loss from cerrado grasslands and productive lands could be varying from 134 Tg C to 180 Tg C, while in this study we quantified a C loss of 57.5 Tg.

Second, this study have not accounted for the effects of selective logging and repeated fires. It has been estimated that 8–15% of the original biomass could be removed by legal and illegal selective logging in the Amazon and this pool would have a lifetime of approximately 30 years [*Alencar et al.*, 2006; *Aguiar et al.*, 2012]. The effects of selective logging until the year 2000 are probably captured in the AGB estimates used in this study [*Saatchi et al.*, 2012] but could have some implications on the carbon stocks of forests closer to more recent deforested areas. Repeated fires over burned areas would increase immediate emissions (gross emissions) and decrease the time for decomposing the AGB dead material, but in terms of the quantified committed emissions, it would have no effect.

The relationship between burned areas and active fire pixels is likely to misrepresent the spatial pattern or extent of the fire occurrence, especially for small burned areas. In open vegetation types, such as cerrado and productive lands, fires spread quickly, and active fires can be potentially extinguished between two MODIS/Terra satellite overpasses: active fires were detected between 1 h and 3 h in the morning and 13 h and 15 h in the afternoon in the study area (Greenwich mean time).

The Global Fire Emission Database version 3 (GFED3) monthly C emission product [*van der Werf et al.*, 2010] estimated a carbon emission of 190 Tg for the same period in Mato Grosso state, a value 2.5 times higher than our highest estimate. The GFED3 uses 10 parameters with a spatial resolution from 500 m to 2° as an input for the Carnegie‐Ames‐Stanford approach‐GFED3 model. The main factors that may explain these differences are likely to be associated with three components of the GFED3 model, and the low spatial resolution of some of the input data, which can underestimate or overestimate different land use and land cover types. First, the difference in the spatial variability of the biomass map used by *van der Werf et al.* [2010]—GFED3—and *Saatchi et al.* [2011] used in our study.

Second, GFED3 takes into account the combustion of leaves, litter, and coarse wood debris in forests, not accounted for in this study. According to *Chave et al.* [2010], the average values of annual production of litterfall and fine litter for cerrado and forest, derived from field plots in Mato Grosso, are 1.211 Mg ha^−1^ yr^−1^ and 12.12 Mg ha^−1^ yr^−1^, respectively. Assuming that no decomposition has occurred until the end of the dry season and assuming complete combustion of these materials, the maximum contribution from litterfall in our study for the C emissions in the burned areas would be 12.8 Tg. Coarse wood debris stocks in forests can comprise up to 33% of the biomass of trees >10 cm DBH [*Baker et al.*, 2007]. Assuming that these maximum values and the combustion completeness of this material vary between 0.4 and 0.6, in deforested lands [*van der Werf et al.*, 2010], we estimate a higher boundary of emissions from coarse woody debris between 15 and 23 Tg C for old‐growth forests and between 16 and 24 Tg C for old‐growth cerrado in MT. Therefore, by summing the C emissions from litterfall with our estimates, the total would be 99.9 Tg to 115.9 Tg of C.

Third, the GFED3 model uses fire persistence as a proxy for indicating deforestation fires and thus generates higher emissions than forest fires solely. During extreme droughts, the increase in forest flammability would allow fire flames to reach the canopy of stand forests, and it could lead to persistent fire pixel detection not related to deforestation. Therefore, emissions from these fires could be lower than deforestation fires leading to higher estimates in the GFED3 data in relation to our approach.

A recent quantification of carbon emissions in Amazonia using atmospheric measurements reveals that 0.51 ± 0.12 Pg C were emitted from fires in 2010 [*Gatti et al.*, 2014]. From this total, we estimate that Mato Grosso could be potentially responsible for at least 6.6% to 15.4% of the emissions, and by including the estimates from litterfall and coarse wood debris, Mato Grosso fires would be responsible for 19.6% to 22.5%. Old‐growth forest fires in the Brazilian Legal Amazon would contribute at least with 2.9% (2.3% to 3.5%) of the total emissions in 2010.

## 5 Conclusions

Brazil's National Plan on Climate Change (NPCC; Decree No. 6263 of 21 November 2008) [*NPCC‐Interministerial Committee on Climate Change*, 2008] established national targets for reducing the deforestation rates below a baseline of the average deforestation from 1996 to 2005. This represents a decrease in the emission to the atmosphere of 1.3 Pg C from 2006 to 2017 [*NPCC*, 2008], approximately 0.11 Pg C yr^−1^. The total C loss due to fires in Mato Grosso state quantified in this study (0.085 ± 0.033 Pg C) represents 77% (47% to 107%) of the national annual target for reductions in Amazonia. By considering only old‐growth forest fires in the Brazilian Legal Amazon, the total carbon loss corresponds to 86% (68.2% to 103%) of the annual target. Considering that fires in intact vegetation (primary forests and cerrado) in Mato Grosso were responsible for approximately 78% of the emissions, more effort in monitoring uncontrolled fires in these protected areas should be a key policy strategy for achieving emission reductions in the long term in order to minimize climate change. A possible solution for monitoring large‐scale fires in Brazil is the development of site‐specific fire risk and fire propagation models at a spatial scale that can support decision makers.

## Supporting information

Texts S1–S5 and Figures S1 and S2

Table S1

Table S2

Tables S3–S5

## References

[gbc20344-bib-0001] Aguiar, A. P. D. , et al. (2012), Modeling the spatial and temporal heterogeneity of deforestation‐driven carbon emissions: The INPE‐EM framework applied to the Brazilian Amazon, Global Chang. Biol., 18, 3346–3366, doi:10.1111/j.1365-2486.2012.02782.x.

[gbc20344-bib-0122] Alencar, A. , D. Nepstad , P. Moutinho (2005), Carbon emissions associated with forest fires in Brazil, in Tropical Deforestation and Climate Change. [Available at http://www.edfclimatecorps.net/sites/default/files/4930_TropicalDeforestation_and_ClimateChange.pdf#page=23.]

[gbc20344-bib-0002] Alencar, A. , D. C. Nepstad , and M. C. V. Diaz (2006), Forest understory fire in the Brazilian Amazon in ENSO and non‐ENSO years: Area burned and committed carbon emissions, Earth Interact., 10, 1–17, doi:10.1175/EI150.1.

[gbc20344-bib-0003] Almeida‐Filho, R. , and Y. E. Shimabukuro (2002), Digital processing of a Landsat‐TM time series for mapping and monitoring degraded areas caused by independent gold miners, Roraima state, Brazilian Amazon, Remote Sens. Environ., 79, 42–50.

[gbc20344-bib-0004] Anderson, L. O. , L. E. Oliveira e Cruz de Aragão , A. de Lima , and Y. E. Shimabukuro (2005), Detecção de cicatrizes de áreas queimadas baseada no modelo linear de mistura espectral e imagens índice de vegetação utilizando dados multitemporais do sensor MODIS/Terra no estado do Mato Grosso, Amazônia brasileira, Acta Amaz., 35, 445–456.

[gbc20344-bib-0006] Anderson, L. O. , M. Trivedi , J. Queiroz , L. Aragão , J. A. Marengo , C. Young , and P. Meir (2011), Counting the costs of the 2005 Amazon drought: A preliminary assessment, in Ecosystem Services for Poverty Alleviation in Amazonia, edited by Meir P. et al., pp. 96–108, Global Canopy Programme and Univ. of Edinburgh, Edinburgh.

[gbc20344-bib-0007] Aragão L. E. O. C. , and Y. E. Shimabukuro (2010), The incidence of fire in Amazonian forests with implications for REDD, Science, 328, 1275–1278. [Available at http://www.sciencemag.org/content/328/5983/1275.abstract.] 20522775 10.1126/science.1186925

[gbc20344-bib-0008] Aragão, L. E. O. C. , Y. Malhi , R. M. Roman‐Cuesta , S. Saatchi , L. Anderson , and Y. E. Shimabukuro (2007), Spatial patterns and fire response of recent Amazonian droughts, Geophys. Res. Lett., 34 L07701, doi:10.1029/2006GL028946.

[gbc20344-bib-0009] Aragão, L. E. O. C. , B. Poulter , J. B. Barlow , L. O. Anderson , Y. Malhi , S. Saatchi , O. L. Phillips , and E. Gloor (2014), Environmental change and the carbon balance of Amazonian forests, Biol. Rev., 89, 913–931, doi:10.1111/brv.12088. 25324039 10.1111/brv.12088

[gbc20344-bib-0010] Araújo, T. M. , J. A. Carvalho , N. Higuchi , A. C. P. Brasil , and A. L. A. Mesquita (1999), A tropical rainforest clearing experiment by biomass burning in the state of Pará, Brazil, Atmos. Environ., 33(13), 1991–1998.

[gbc20344-bib-0012] Baccini, A. , et al. (2012), Estimated carbon dioxide emissions from tropical deforestation improved by carbon‐density maps, Nat. Clim. Change, 2, 182–185, doi:10.1038/nclimate1354.

[gbc20344-bib-0013] Baker, T. R. , E. N. H. Coronado , O. L. Phillips , J. Martin , G. M. van der Heijden , M. Garcia , and J. S. Espejo (2007), Low stocks of coarse woody debris in a southwest Amazonian forest, Oecologia, 152(3), 495–504. 17333287 10.1007/s00442-007-0667-5

[gbc20344-bib-0014] Balch, J. K. (2014), Atmospheric science: Drought and fire change sink to source, Nature, 506, 41–42, doi:10.1038/506041a. 24499913 10.1038/506041a

[gbc20344-bib-0015] Balch J. K. , D. C. Nepstad , L. M. Curran , P. M. Brando , O. Portela , P. Guilherme , J. D. Reuning‐Scherer , and O. de Carvalho Jr. (2011), Size, species, and fire behavior predict tree and liana mortality from experimental burns in the Brazilian Amazon, Forest Ecol. Manage., 261, 68–77. [Available at http://www.sciencedirect.com/science/article/pii/S037811271000561X.]

[gbc20344-bib-0016] Barbosa R. I. , and P. M. Fearnside (1999), Incêndios na Amazônia brasileira: Estimativa da emissão de gases do efeito estufa pela queima de diferentes ecossistemas de Roraima na passagem do evento “El Niño” 1997/98, Acta Amaz., 29, 513–534. [Available at https://acta.inpa.gov.br/fasciculos/29-4/PDF/v29n4a02.pdf.]

[gbc20344-bib-0017] Barlow J. , and C. A. Peres (2008), Fire‐mediated dieback and compositional cascade in an Amazonian forest, Philos. Trans. R Soc. B Biol. Sci., 363, 1787–1794. [Available at http://rstb.royalsocietypublishing.org/content/363/1498/1787.abstract.] 10.1098/rstb.2007.0013 PMC2373873 18267911

[gbc20344-bib-0018] Barlow, J. , C. A. Peres , B. O. Lagan , and T. Haugaasen (2003), Large tree mortality and the decline of forest biomass following Amazonian wildfires, Ecol. Lett., 6, 6–8, doi:10.1046/j.1461-0248.2003.00394.x.

[gbc20344-bib-0019] Berenguer, E. , J. Ferreira , T. A. Gardner , L. E. O. C. Aragão , P. B. De Camargo , C. E. Cerri , M. Durigan , R. C. D. Oliveira , I. C. G. Vieira , and J. Barlow (2014), A large‐scale field assessment of carbon stocks in human‐modified tropical forests, Global Change Biol., 20, 3713–3726, doi:10.1111/gcb.12627. 10.1111/gcb.12627 24865818

[gbc20344-bib-0123] Bins, L. S. , G. J. Erthal , and L. M. G. Fonseca (1993), Um Método de Classificação Não Supervisionada por Regiões, in SIBGRAPI VI, Recife, PE, Anais, pp. 65–68.

[gbc20344-bib-0020] Bloomfield, P. (2014), Statistics 430 and Statistics 514 course, material on‐line. [Available at http://www.stat.ncsu.edu/people/bloomfield/courses/st430-514/, NC State University Department.]

[gbc20344-bib-0021] Brando, P. M. , D. C. Nepstad , J. K. Balch , B. Bolker , M. C. Christman , M. Coe , and F. E. Putz (2012), Fire‐induced tree mortality in a neotropical forest: The roles of bark traits, tree size, wood density and fire behavior, Global Chang Biol., 18, 630–641, doi:10.1111/j.1365-2486.2011.02533.x.

[gbc20344-bib-0022] Cai, W. , et al. (2014), Increasing frequency of extreme El Niño events due to greenhouse warming, Nat. Clim. Change, 4, 111–116, doi:10.1038/nclimate2100.

[gbc20344-bib-0023] Cardozo, F. S. , G. Pereira , Y. E. Shimabukuro , and E. C. Moraes (2014), Analysis and assessment of the spatial and temporal distribution of burned areas in the Amazon forest, Remote Sens., 6(9), 8002–8025.

[gbc20344-bib-0024] Carroll, R. J. , and D. Ruppert (1988), Transformation and Weighting in Regression, pp. 161–173, Chapman and Hall, New York.

[gbc20344-bib-0025] Carvalho, J. A. , N. Higuchi , T. M. Araújo , and J. C. Santos (1998), Combustion completeness in a rainforest clearing experiment in Manaus, Brazil, J. Geophys. Res., 103(D11), 13,195–13,199, doi:10.1029/98JD00172.

[gbc20344-bib-0026] Chambers, J. Q. , N. Higuchi , J. P. Schimel , L. V. Ferreira , and J. M. Melack (2000), Decomposition and carbon cycling of dead trees in tropical forests of the central Amazon, Oecologia, 122, 380–388, doi:10.1007/s004420050044. 10.1007/s004420050044 28308289

[gbc20344-bib-0027] Chang, D. , and Y. Song (2009), Comparison of L3JRC and MODIS global burned area products from 2000 to 2007, J. Geophys. Res., 114 D16106, doi:10.1029/2008JD011361.

[gbc20344-bib-0028] Chave J. , et al. (2010), Regional and seasonal patterns of litterfall in tropical South America, Biogeosciences, 7, 43–55. [Available at http://www.biogeosciences.net/7/43/2010/.]

[gbc20344-bib-0029] Chen, Y. , J. T. Randerson , D. C. Morton , R. S. DeFries , G. J. Collatz , P. S. Kasibhatla , L. Giglio , Y. Jin , and M. E. Marlier (2011), Forecasting fire season severity in South America using sea surface temperature anomalies, Science, 334(6057), 787–791, doi:10.1126/science.1209472. 22076373 10.1126/science.1209472

[gbc20344-bib-0030] Chuvieco, E. , et al. (2008), Global burned‐land estimation in Latin America using MODIS composite data, Ecol. Appl., 18, 64–79, doi:10.1890/06-2148.1. 18372556 10.1890/06-2148.1

[gbc20344-bib-0032] Cox, P. M. , P. P. Harris , C. Huntingford , R. A. Betts , M. Collins , C. D. Jones , T. E. Jupp , J. A. Marengo , and C. A. Nobre (2008), Increasing risk of Amazonian drought due to decreasing aerosol pollution, Nature, 453, 212–215, doi:10.1038/nature06960. 18464740 10.1038/nature06960

[gbc20344-bib-0033] de Mendonça M. J. C. , M. d. C. Vera Diaz , D. Nepstad , R. Seroa da Motta , A. Alencar , J. C. Gomes , and R. A. Ortiz (2004), The economic cost of the use of fire in the Amazon, Ecol. Econ., 49, 89–105. [Available at http://www.sciencedirect.com/science/article/pii/S0921800904000424.]

[gbc20344-bib-0035] Espinoza, J. C. , J. Ronchail , F. Frappart , W. Lavado , W. Santini , and J. L. Guyot (2013), The major floods in the Amazonas river and tributaries (Western Amazon Basin) during the 1970–2012 period: A focus on the 2012 flood, J. Hydrometeorol., 14, 1000–1008, doi:10.1175/JHM-D-12-0100.1.

[gbc20344-bib-0036] Gatti, L. V. , et al. (2014), Drought sensitivity of Amazonian carbon balance revealed by atmospheric measurements, Nature, 506, 76–80, doi:10.1038/nature12957. 24499918 10.1038/nature12957

[gbc20344-bib-0038] Giglio L. (2010), MODIS collection 5 active fire product user's guide. [Available at http://modis-fire.umd.edu/Documents/MODIS_Fire_Users_Guide_2.4.pdf.]

[gbc20344-bib-0039] Giglio L. , G. R. van der Werf , J. T. Randerson , G. J. Collatz , and P. Kasibhatla (2006), Global estimation of burned area using MODIS active fire observations, Atmos. Chem. Phys., 6, 957–974. [Available at http://www.atmos-chem-phys.net/6/957/2006/.]

[gbc20344-bib-0042] Instituto Nacional de Pesquisas Espaciais (INPE) (2015), PRODES: Assessment of Deforestation in Brazilian Amazonia. [Available at www.obt.inpe.br/prodes/index.html.]

[gbc20344-bib-0043] Jain, A. K. (2007), Global estimation of CO emissions using three sets of satellite data for burned area, Atmos. Environ., 41, 6931–6940.

[gbc20344-bib-0044] Kauffman J. B. , D. L. Cummings , and D. E. Ward (1994), Relationships of fire, biomass and nutrient dynamics along a vegetation gradient in the Brazilian cerrado, J. Ecol., 82, 519–531. [Available at http://www.jstor.org/stable/2261261.]

[gbc20344-bib-0045] Lima, A. , T. S. F. Silva , L. E. O. C. Aragão , R. M. de Feitas , M. Adami , A. R. Formaggio , and Y. E. Shimabukuro (2012), Land use and land cover changes determine the spatial relationship between fire and deforestation in the Brazilian Amazon, Appl. Geogr., 34, 239–246.

[gbc20344-bib-0046] Macedo M. N. , R. S. DeFries , D. C. Morton , C. M. Stickler , G. L. Galford , and Y. E. Shimabukuro (2012), Decoupling of deforestation and soy production in the southern Amazon during the late 2000s, Proc. Natl. Acad. Sci. U.S.A., 109, 1341–1346. [Available at http://www.pnas.org/content/early/2012/01/06/1111374109.abstract.] 22232692 10.1073/pnas.1111374109 PMC3268292

[gbc20344-bib-0047] Malhi Y. , J. T. Roberts , R. A. Betts , T. J. Killeen , W. Li , and C. A. Nobre (2008), Climate change, deforestation, and the fate of the Amazon, Science, 319, 169–172. [Available at http://www.sciencemag.org/content/319/5860/169.abstract.] 18048654 10.1126/science.1146961

[gbc20344-bib-0048] Marengo, J. A. (2009), Long‐term trends and cycles in the hydrometeorology of the Amazon Basin since the late 1920s, Hydrol. Process, 23, 3236–3244, doi:10.1002/hyp.7396.

[gbc20344-bib-0049] Marengo, J. A. , J. Tomasella , L. M. Alves , W. R. Soares , and D. A. Rodriguez (2011), The drought of 2010 in the context of historical droughts in the Amazon region, Geophys. Res. Lett., 38 L12703, doi:10.1029/2011GL047436.

[gbc20344-bib-0050] Martini P. R. , V. Duarte , and Y. E. Shimabukuro (2012), Panamazônia II project. [Available at http://www.dsr.inpe.br/laf/panamazonia.html. Instituto Nacional de Pesquisas Espaciais (INPE).]

[gbc20344-bib-0124] McGregor, S. , A. Timmermann , M. F. Stuecker , M. H. England , M. Merrifield , F.‐F. Jin , and Y. Chikamoto (2014), Recent Walker circulation strengthening and Pacific cooling amplified by Atlantic warming, Nat. Clim. Change, 4, 888–892, doi:10.1038/nclimate2330.

[gbc20344-bib-0051] Morton, D. C. , R. Defries , J. Nagol , C. M. Souza Jr. , E. S. Kasischke , G. C. Hurtt , and R. Dubayah (2011), Mapping canopy damage from understory fires in Amazon forests using annual time series of Landsat and MODIS data, Remote Sens. Environ., 115, 1706–1720.

[gbc20344-bib-0052] Morton D. C. , Y. Le Page , R. DeFries , G. J. Collatz , and G. C. Hurtt (2013), Understorey fire frequency and the fate of burned forests in southern Amazonia, Philos. Trans. R Soc. B Biol. Sci., 368 [Available at http://rstb.royalsocietypublishing.org/content/368/1619/20120163.abstract.] 10.1098/rstb.2012.0163 PMC3638429 23610169

[gbc20344-bib-0053] NIST/SEMATECH (2015), e‐Handbook of Statistical Methods. [Available at http://www.itl.nist.gov/div898/handbook/, accessed on 5th June.]

[gbc20344-bib-0054] NPCC‐Interministerial Committee on Climate Change (2008), National Plan on Climate Change‐Decree No. 6263. Brasilia. [Available at http://www.mma.gov.br/estruturas/imprensa/_arquivos/96_11122008040728.pdf.]

[gbc20344-bib-0055] Oliveras, I. , S. T. Meirelles , V. L. Hirakuri , C. R. Freitas , H. S. Miranda , and V. R. Pivello (2013), Effects of fire regimes on herbaceous biomass and nutrient dynamics in the Brazilian savanna, Int. J. Wildland Fire, 22, 368–380, doi:10.1071/WF10136.

[gbc20344-bib-0056] Oliveras, I. , L. O. Anderson , and Y. Malhi (2014), Application of remote sensing to understanding fire regimes and biomass burning emissions of the tropical Andes, Global Biogeochem. Cycles, 28, 480–496, doi:10.1002/2013GB004664.

[gbc20344-bib-0058] Roy D. P. , L. Boschetti , and C. O. Justice , Ju J. (2008), The collection 5 MODIS burned area product—Global evaluation by comparison with the MODIS active fire product, Remote Sens. Environ., 112, 3690–3707. [Available at http://www.sciencedirect.com/science/article/pii/S0034425708001752.]

[gbc20344-bib-0059] Saatchi, S. S. , R. A. Houghton , R. C. dos Santos Alvalá , J. V. Soares , and Y. Yu (2007), Distribution of aboveground live biomass in the Amazon Basin, Global Change Biol., 13, 816–837, doi:10.1111/j.1365-2486.2007.01323.x.

[gbc20344-bib-0060] Saatchi S. S. , et al. (2011), Benchmark map of forest carbon stocks in tropical regions across three continents, Proc. Natl. Acad. Sci. U.S.A., 108, 9899–9904. [Available at http://www.pnas.org/content/108/24/9899.abstract.] 21628575 10.1073/pnas.1019576108 PMC3116381

[gbc20344-bib-0061] Saatchi S. , S. Asefi‐Najafabady , Y. Malhi , L. E. O. C. Aragão , L. O. Anderson , R. B. Myneni , and R. Nemani (2012), Persistent effects of a severe drought on Amazonian forest canopy, Proc. Natl. Acad. Sci. U.S.A., 110, 565–570. [Available at http://www.pnas.org/content/early/2012/12/19/1204651110.abstract.] 23267086 10.1073/pnas.1204651110 PMC3545782

[gbc20344-bib-0062] Satyamurty, P. , C. P. W. Costa , A. O. Manzi , and L. A. Candido (2013), A quick look at the 2012 record flood in the Amazon Basin, Geophys. Res. Lett., 40, 1396–1401, doi:10.1002/grl.50245.

[gbc20344-bib-0063] Schroeder, W. , E. Prins , L. Giglio , I. Csiszar , C. Schmidt , J. Morisette , and D. Morton (2008), Validation of GOES and MODIS active fire detection products using ASTER and ETM+ data, Rem. Sens. Environ., 112, 2711–2726.

[gbc20344-bib-0164] Shimabukuro, Y. E. , G. T. Batista , E. M. K. Mello , J. C. Moreira , and V. Duarte (1998), Using shade fraction image segmentation to evaluate deforestation in landsat thematic mapper images of the Amazon region, Intl. J. Remote Sens., 19(3), 535–541.

[gbc20344-bib-0064] Shimabukuro, Y. E. , V. Duarte , E. Arai , R. M. Freitas , A. Lima , D. Valeriano , I. F. Brown , and M. L. R. Maldonado (2009), Fraction images derived from Terra MODIS data for mapping burnt areas in Brazilian Amazonia, Int. J. Remote Sens., 30, 1537–1546, doi:10.1080/01431160802509058.

[gbc20344-bib-0065] Shimabukuro, Y. E. , V. Duarte , E. Arai , R. M. Freitas , P. R. Martini , and A. Lima (2010), Monitoring land cover in Acre State, western Brazilian Amazonia, using multitemporal remote sensing data, Int. J. Image Data Fusion, 1, 325–335, doi:10.1080/19479832.2010.505177.

[gbc20344-bib-0067] Smith, L. T. , L. E. O. C. Aragão , C. E. Sabel , and T. Nakaya (2014), Drought impacts on children's respiratory health in the Brazilian Amazon, Sci. Rep., 4, doi:10.1038/srep03726. 10.1038/srep03726 PMC3893650 24430803

[gbc20344-bib-0068] Song, X.‐P. , C. Huang , S. S. Saatchi , M. C. Hansen , and J. R. Townshend (2015), Annual carbon emissions from deforestation in the Amazon Basin between 2000 and 2010, PLoS One, 10(5), e0126754, doi:10.1371/journal.pone.0126754. 10.1371/journal.pone.0126754 PMC4423949 25951328

[gbc20344-bib-0069] Tan, J. , C. Jakob , W. B. Rossow , and G. Tselioudis (2015), Increases in tropical rainfall driven by changes in frequency of organized deep convection, Nature, 519, 451–454, doi:10.1038/nature14339. 25810207 10.1038/nature14339

[gbc20344-bib-0070] van der Werf G. R. , D. C. Morton , R. S. DeFries , L. Giglio , J. T. Randerson , G. J. Collatz , and P. S. Kasibhatla (2009), Estimates of fire emissions from an active deforestation region in the southern Amazon based on satellite data and biogeochemical modelling, Biogeosciences, 6, 235–249. [Available at http://www.biogeosciences.net/6/235/2009/.]

[gbc20344-bib-0071] van der Werf G. R. , J. T. Randerson , L. Giglio , G. J. Collatz , M. Mu , P. S. Kasibhatla , D. C. Morton , R. S. DeFries , Y. Jin , and T. T. van Leeuwen (2010), Global fire emissions and the contribution of deforestation, savanna, forest, agricultural, and peat fires (1997–2009), Atmos. Chem. Phys., 10, 11,707–11,735. [Available at http://www.atmos-chem-phys.net/10/11707/2010/.]

[gbc20344-bib-0072] Ward D. E. , and C. C. Hardy (1991), Smoke emissions from wildland fires, Environ. Int., 17, 117–134. [Available at http://www.sciencedirect.com/science/article/pii/0160412091900958.]

[gbc20344-bib-0073] Wolfe R. E. , M. Nishihama , A. J. Fleig , J. A. Kuyper , D. P. Roy , J. C. Storey , and F. S. Patt (2002), Achieving sub‐pixel geolocation accuracy in support of MODIS land science, Remote Sens. Environ., 83, 31–49. [Available at http://www.sciencedirect.com/science/article/pii/S0034425702000858.]

[gbc20344-bib-0074] York, D. , N. Evensen , M. Martinez , and J. Delgado (2004), Unified equations for the slope, intercept, and standard errors of the best straight line, Am. J. Phys., 72(3), doi:10.1119/1.1632486.

