# Supplementary material for: Disentangling the contribution of multiple land covers to fire‐mediated carbon emissions in Amazonia during the 2010 drought
Source: Global Biogeochem Cycles. 2015 Oct 22;29(10):1739–53. doi: 10.1002/2014GB005008 (PMC4994379; doi:10.1002/2014GB005008)
Supplement: Supplementary file 1 — Texts S1–S5 and Figures S1 and S2 [file GBC-29-1739-s001.doc]

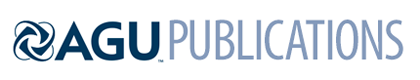


*Global Biogeochemical Cycles*

Supporting Information for

**DISENTANGLING THE CONTRIBUTION OF MULTIPLE LAND COVERS TO FIRES-MEDIATED CARBON EMISSIONS IN AMAZONIA DURING THE 2010 DROUGHT**

Liana Oighenstein Anderson1,2,3*, Luiz E. O. C. Aragão3,4, Manuel Gloor5, Egídio Arai3, Marcos Adami3, Sassan Saatchi6,7, Yadvinder Malhi2, Yosio E. Shimabukuro3, Jos Barlow8, Erika Berenguer8, Valdete Duarte3

1 National Center for Monitoring and Early Warning of Natural Disasters – Cemaden, São José dos Campos, Brazil, 12.247-016

2 Environmental Change Institute, University of Oxford, Oxford, OX1 3QY, UK

3 Remote Sensing Division, National Institute for Space Research, São José dos Campos, Brazil, 12227-010

4College of Life and Environmental Sciences, University of Exeter, Exeter, EX4 4RJ, UK

5School of Geography, University of Leeds, Leeds, LS2 9JT, UK

6Jet Propulsion Laboratory, California Institute of Technology, Pasadena, CA 91109

7Institute of Environment, University of California, Los Angeles, CA 90045

8Lancaster Environment Centre, Lancaster University, Lancaster, LA1 4YQ, UK

**Contents of this file**

Text S1 to S5

Figures S1 to S2

Tables S1 to S5

**Additional Supporting Information (Files uploaded separately)**

Table S1. Error matrix comparing the Mato Grosso classification with the reference points. Overall accuracy is referred as “P”.

Table S2. Above Ground Biomass (AGB) based on Saatchi et al., 2011 of the land cover classes (LCC).

Table S3. Above ground biomass stocks, carbon loss and emissions based on Saatchi et al., 2007 and Baccini et al., 2012, referred in the table below as S7 and B12, respectively.

**Introduction**

This document includes additional text for providing more information on the (S1) Land cover map, (S2) Above ground biomass map, (S3) Comparison of the above ground biomass datasets, (S4) Burn scar mapping and the accuracy assessment. Also, two additional figure (S1 and S2) are included here. Finally 5 supporting tables (S1 to S5) are provided, and three of them (Table S1 to Table S3) were uploaded in a separate file due to its length.

Text S1. Land cover map

The PANAMAZONIA land cover map was developed using satellite images from 1980 (Landsat Multispectral Scanner System (MSS)), 1990 (Landsat Thematic Mapper TM)), 2000 (Landsat Enhanced Thematic Mapper Plus (ETM+)), 2009 and 2010 (Terra Moderate Resolution Imaging Spectroradiometer (MODIS)), based on the methodology developed by Shimabukuro et al, 2010.

In summary, the initial map was derived from the Landsat/ETM+, year 2000, with a high quality orthorectification provided by the NASA Geocover product, resampled from 30 m spatial resolution to 100 m spatial resolution. Then, the original map was updated for 1990, using the Landsat Thematic Mapper TM resampled from 30 m spatial resolution to 100 m spatial resolution. The next step was to update the map for the year 1980 using the Landsat Multispectral Scanner (MSS) images resampled from 80 m to 100 m spatial resolution. Thus, the MSS, TM and ETM+ were resampled to 100 m spatial resolution for bringing the dataset to a spatial resolution closer to the MODIS product. The Landsat datasets were used for analyzing the land use and land cover changes during the 20 years (1980–2000) time period. Following, MODIS surface reflectance images (product MOD09GQ collection 5, 250m spatial resolution) obtained in 2009 and 2010 dry season were used to map the cumulative deforestation occurring between 2000 and 2009-2010. Before performing this task the 100 m spatial resolution land cover map was resampled to be compatible to MODIS 250 m spatial resolution datasets. The agreement between land cover classes in the multitemporal classification is assured by the high quality georeferencing system of MODIS and the agreement with the 2000 Landsat orthorectified data available from NASA. For more details in the methods, please refer to Shimabukuro et al., 2010.

An accuracy assessment of the land cover map was performed based on an evaluation of the derived land cover map against an independent dataset of MSS and Landsat images covering 1980 to 2010, totalling 191 Landsat images and 43 MSS images. In order to perform a confusion matrix (ESM Table 1) and generate the indices user’s and producer’s accuracy, overall classification accuracy (P), confidence interval for P and the Kappa coefficient of agreement, 101 random points were evaluated. The overall accuracy of the map was 80%. Course resolution datasets presents mixed pixels, particularly where land cover mosaic is complex. Therefore, by excluding the random points at class boundaries (11 points) due to the scale difference from the reference data (Landsat with 30 m spatial resolution) (Fuller et al., 1994), the overall accuracy increases to 90% (95% confidence interval for P: 85%-95%) and the kappa coefficient increases to 90%.

A complete description of the methodology can be found in:

Shimabukuro YE, Duarte V, Arai E, Freitas RM, Martini PR, Lima A. 2010 Monitoring land cover in Acre State, western Brazilian Amazonia, using multitemporal remote sensing data. International Journal of Image and Data Fusion, 1(4), 325-335. (DOI: 10.1080/19479832.2010.505177)

Fuller, R. M., Groom, G. B., & Jones, A. R. 1994 The land cover map of Great Britain: an automated classification of Landsat Thematic Mapper data. Photogrammetric Engineering and Remote Sensing, 60, 553–562.

More information about the PANAMAZONIA project and the Mato Grosso land cover maps and the 2010 burned area map coupled with Google Earth can be found at:

http://www.dsr.inpe.br/laf/panamazonia/dados.html

***Text S2. Above Ground Biomass map***

To quantify the biomass for the land cover classes mapped for the year 2010, we maintained the 2000’s biomass values for classes that have not changed (intact vegetation, pre-2000 land cover dynamics). However, for the classes that have changed from 2000 to 2010 (productive areas cleared in this period) we used the average biomass values of the specific land cover class in 2000 with similar land cover change history to update the AG biomass values for 2010. For example, the class “forests cleared between 2001 and 2009” present the average biomass values from the land cover class “Forest cleared in 1990” from the map for the year 2000. Thus, the biomass values applied to these areas correspond to deforested areas with approximately 10 years of conversion. To be conservative, we grouped the classes: (1) forest regrowth with less than 20 years, (2) forest regrowth with less than 10 years and (3) deforestation on forest regrowth and applied the biomass values from the land cover class “regrowth with less than 10-years” derived from the map for the year 2000. Similarly, the Cerrado area cleared between 2001 and 2010 received a biomass value derived from the land cover class “Cerrado cleared in 1990” from the land cover map for the year 2000. For the Cerrado regrowth in 2010, we used the values for this same class from the map 2000 (ESM Table 2). The table below presents the detailed information for the pixel-based average AGB for each land cover type, assuming average AGB derived from the original map (Saatchi et al., 2011) for each land cover type. In order to provide an uncertainty in relation to the values presented in this table, the Error map from Saatchi et al. (2011) were also estimated per land cover type.

***Text S3. Comparison of the above ground biomass datasets***

Three datasets were used in this study in order to discuss the differences in carbon stocks and emissions associated with the input biomass data from the main manuscript are Saatchi et al., 2007 (average values) and Baccini et al., 2012, referred in the tables below as S7 and B12, respectively.

***Text S4. Burn scar mapping and accuracy assessment***

Although the images derived from MOD09-GQ/Q1 have an original spatial resolution of 231.7 meters2 and MOD09-GA/A1 have a spatial resolution of 463.3 meters2 in size, all bands were resampled to 250m2 spatial resolution using a nearest neighbour algorithm and standardized to the geotiff format in geographic projection with WGS 84 Datum using the MODIS Reprojection Tool (MRT, 2011).

Similar to active fire detection, the burn scar mapping can underestimate the burnt area when the type of burning does not generate a scar detectable at the pixel resolution (e.g. the burning of piled wood debris) or when rainy events occur within the interval between image dates acquisition. The influence of the latter factor is likely to be reduced because images were acquired only for the dry season.

MODIS Reprojection Tool - MRT - User´s manual (2011). Available: https://lpdaac.usgs.gov/tools/modis_reprojection_tool.

We acquired Landsat 5 (TM) and 7 (ETM+) images for areas mapped with low confidence by MODIS to improve the maps. These images were used for cross-checking the burned area maps and for guiding the interpreter’s final edition of the burn scars. This procedure was used to correct for misclassification of water bodies, cloud shadows and areas with low confidence, minimizing commission and omission errors.

As one of the major challenges in mapping burn scars is the detection of under canopy fires, in this study we have quantified separately the accuracy of mapping burn scars in forests and non-forests (all other land cover types grouped) areas. We used a stratified random sampling based on the following design: first, the study region was stratified into four distinct classes (burned forest, unburned forests, burned areas and unburned areas). Then, for each one of these classes 150 random points were generated, totalizing 600 random points in order to produce user’s accuracy within a 95% confidence interval. The detailed method is presented in Anderson et al. (under review at Remote Sensing).

***Text S5. References for the data used for Equation 1***

Alencar A, Nepstad D, Vera Diaz MdC. 2006 Forest understory fire in the Brazilian Amazon in ENSO and non-ENSO years: Area burned and committed carbon emissions. Earth Interactions, 10 (6), 1-17.

Barbosa R I, Fearnside P M. 1999. Incêndios na Amazônia brasileira: estimativa da emissão de gases do efeito estufa pela queima de diferentes ecossistemas de Roraima na passagem do evento "El Niño" (1997/98). Acta Amazonica 29:513-534.

Barlow J, Peres CA, Lagan BO, Haugaasen T. 2003. Large tree mortality and the decline of forest biomass following Amazonian wildﬁres. Ecology Letters, 6, 6–8.

Cochrane M A, Schulze M D. 1999. Fire as a recurrent event in tropical forests of the eastern Amazon: effects on forest structure, biomass, and species composition. Biotropica 31(1): 2-16.

Gerwing J J. 2002. Degradation of forests through logging and fire in the eastern Brazilian Amazon. Forest Ecology and Management, 157 (1), pp. 131–141.

Haugaasen T., Barlow J., Peres C A. 2003 Surface wildfires in central Amazonia: short-term impact on forest structure and carbon loss. Forest. Ecol. Mngmt 179, 321–331.

Kauffman J B, Cummings D L, Ward D E. 1994. Relationships of Fire, Biomass and Nutrient Dynamics along a Vegetation Gradient in the Brazilian Cerrado. Journal of Ecology , Vol. 82, No. 3, pp. 519-531


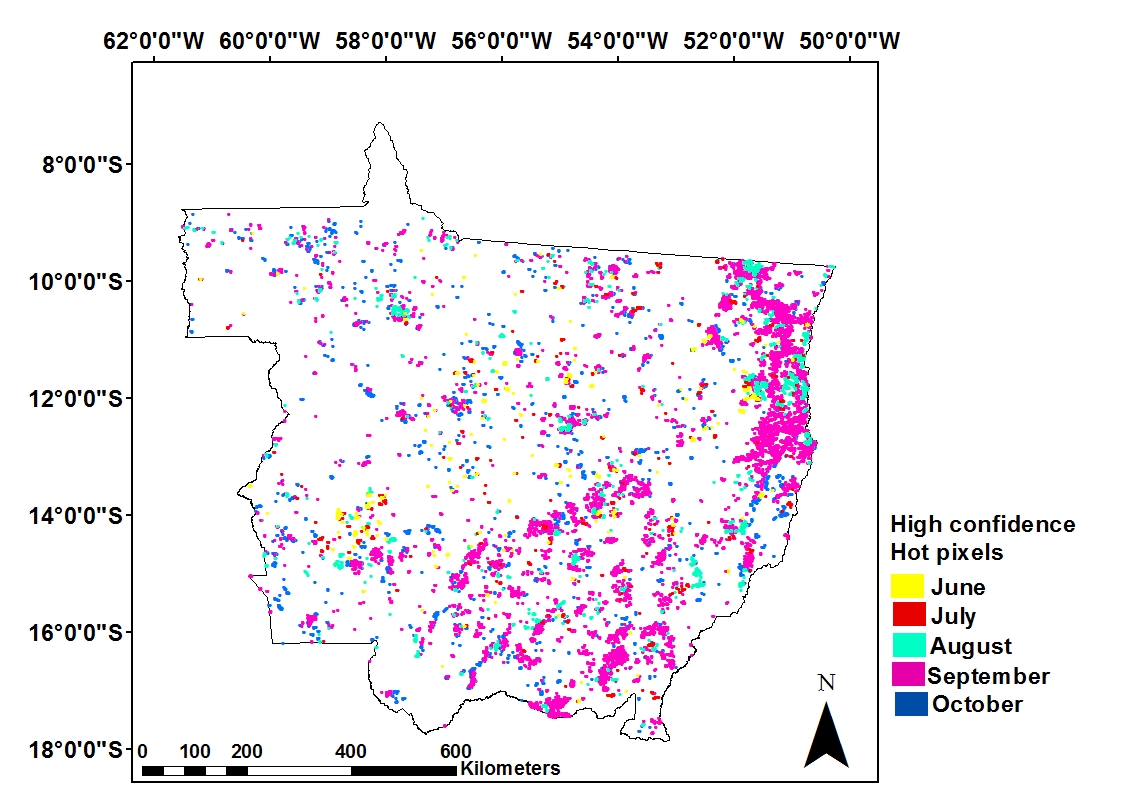


Figure S1. High confidence active fire pixels covering the period analysed - June to 13th October 2010.


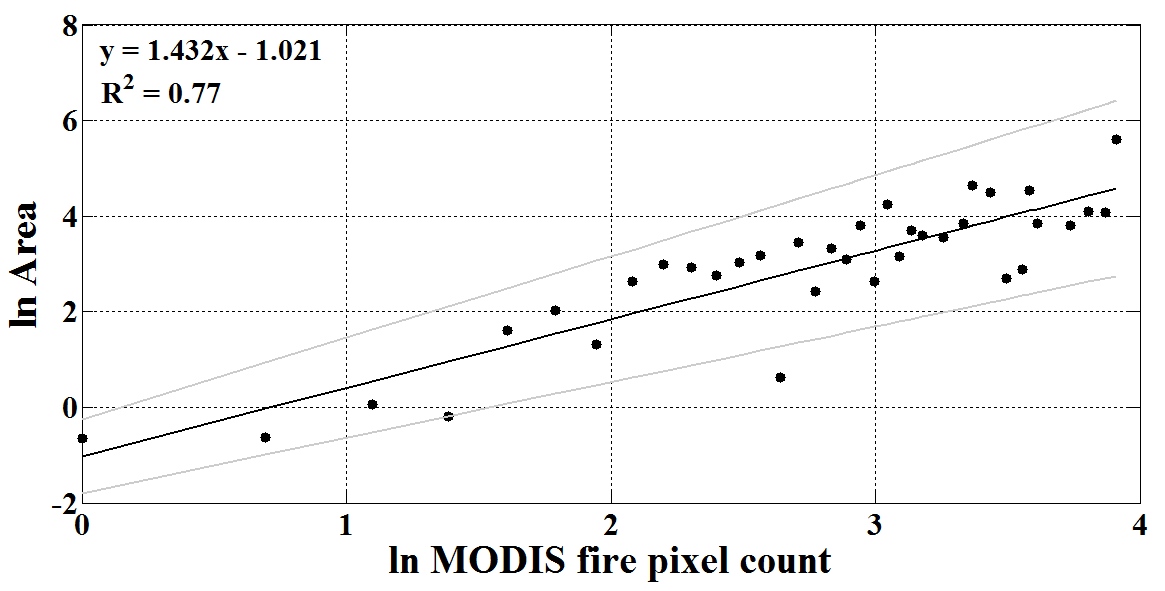


Figure S2. Ordinary Least-Squares Regression (OLS) analysis between primary forests burned area (ha) and fire pixels (count) for Mato Grosso state.

Table S1. Error matrix comparing the Mato Grosso classification with the reference points. Overall accuracy is referred as “P”.

Table S2. Above Ground Biomass (AGB) based on Saatchi et al., 2011 of the land cover classes (LCC).

Table S3. Above ground biomass stocks, carbon loss and emissions based on Saatchi et al., 2007 and Baccini et al., 2012, referred in the table below as S7 and B12, respectively.

Table S4. Accuracy assessment of the burned areas map from June to October, 2010, in Mato Grosso State.

|  | Forest areas | | |  | Non-forest areas | | |
| --- | --- | --- | --- | --- | --- | --- | --- |
|  |  | Confidence interval | |  |  | Confidence interval | |
|  | Estimate | Lower | Upper |  | Estimate | Lower | Upper |
|  |  |  |  |  |  |  |  |
| Class 1 users accuracy | 84.00% | 77.30% | 89.01% |  | 89.00% | 83.38% | 93.33% |
| Class 2 users accuracy | 100.00% | 97.50% | 100.00% |  | 97.33% | 93.34% | 98.96% |
| Class 1 producers accuracy | 100.00% | 100.00% | 100.00% |  | 63.81% | 41.45% | 86.17% |
| Class 2 producers accuracy | 99.16% | 98.86% | 99.47% |  | 99.43% | 99.16% | 99.69% |
| Overall accuracy | 99.20% | 97.67% | 99.48% |  | 96.93% | 93.76% | 98.92% |
| Area error | 0.80% | -0.69% | 1.13% |  | -2.00% | -5.12% | 0.03% |
|  |  |  |  |  |  |  |  |

Table S5. Summary of the data used for developing Equation 1.

| **Reference** | **Location** | **Vegetation type** | **Biomass (Mg ha^-1^)** | |
| --- | --- | --- | --- | --- |
|  | | | Control | Burned |
| Kauffman et al. (1994) | Brasília (15^o^ 51'S, 47^o^63'W) | Cerrado | 7.128 | 2 |
|  |  |  | 7.321 | 0.237 |
|  |  |  | 8.625 | 2.226 |
|  |  |  | 10.031 | 1.604 |
| Cochrane and Schulze (1999) | Tailândia (Central-eastern Para) | Tropical moist evergreen forest | 295 | 270 |
| Haugaasen et al. (2003) | Rio Maró, westernmost Pará (02°44′S; 55°41′W) | River banks forests | 424.3 | 341.7 |
| Barlow et al. (2003) | Rio Maró, westernmost Pará (02°44′S; 55°41′W) | Terra firme forests | 95.4 | 46.8 |
|  |  |  | 91 | 54 |
| Alencar et al. (2006) | Paragominas (NE of Pará) | Dense forest with vines | 485 | 339.5 |
|  | Santana do Araguaia (South of Pará) | Transitional forest | 390 | 273 |
|  | Alta Floresta (North of Mato Grosso) | Open forest | 370 | 259 |
| Alencar et al. (2005) |  |  | 199 | 88 |
| Barbosa and Fearnside (1999) | Roraima | Dense forest | 237 | 202.3 |
