# Supplementary material for: Disentangling the contribution of multiple land covers to fire‐mediated carbon emissions in Amazonia during the 2010 drought
Source: Global Biogeochem Cycles. 2015 Oct 22;29(10):1739–53. doi: 10.1002/2014GB005008 (PMC4994379; doi:10.1002/2014GB005008)
Supplement: Supplementary file 3 — Table S2 [file GBC-29-1739-s003.pdf]

Land cover class	Biomass (Mg ha^{-1})			Total AGB (Mg)
	Mean	Standard deviation	Mean Error	
**Intact vegetation**				
Old growth Forest	212	55.51	33.4	$6.12x10^{9}$
Old growth Cerrado	62.98	57.27	33.6	$1.39x10^{9}$
**Productive lands in the Forest biome**				
Permanent productive for 30 years +	50.53	47.21	33.2	$4.4x10^{7}$
Permanent productive for maximum of 30 years	59.3	55.88	33.5	$1.87x10^{8}$
Permanent productive for maximum of 20 years	78.97	68.96	34	$4.58x10^{8}$
Under consolidation (productive for 10 years or less)	78.97	-		$4.51x10^{8}$
Deforestation in 2010	165	77.36	35.1	$1.36x10^{6}$
**Productive lands in the Cerrado biome**				
Permanent productive for 30 years +	28.98	21.72	31.9	$8.74x10^{7}$
Permanent productive for maximum of 30 years				
Permanent productive for maximum of 20 years	32.77	24.96	32.1	$2.86x10^{8}$
Under consolidation (productive for 10 years or less)	32.77	-		$9.8x10^{7}$
**Regrowth**				
Cerrado	38.84	29.03	32.6	$6.89x10^{6}$
Forest regrowth (less than 20 years)	178.2	76.32	34.4	$2.52x10^{8}$
Forest regrowth (less than 10 years)	172.4	60.19	34.6	$8.58x10^{5}$
Deforestation in 2010 on less than 10 years regrowth	127.8	70.74	33.8	$3.22X10^{7}$
**Total**				**$1.71x10^{10}$**
