# Supplementary material for: Disentangling the contribution of multiple land covers to fire‐mediated carbon emissions in Amazonia during the 2010 drought
Source: Global Biogeochem Cycles. 2015 Oct 22;29(10):1739–53. doi: 10.1002/2014GB005008 (PMC4994379; doi:10.1002/2014GB005008)
Supplement: Supplementary file 4 — Tables S3–S5 [file GBC-29-1739-s004.pdf]

Land cover class	Biomass (Mg ha-1)		Total AGB (Mg)		Total Carbon loss (Mg)		Gross C emission in 2010 (Mg)		Committed CO_2 emission (Mg)	
	S7	B12	S7	B12	S7	B12	S7	B12	S7	B12
**Intact vegetation**										
Old growth Forest	150.6	218.1	$4.36x10^{9}$	$6.28x10^{9}$	$2.15x10^{7}$	$3.07x10^{7}$	$3.37x10^{6}$	$4.81x10^{6}$	$6.00x10^{7}$	$8.56x10^{7}$
Old growth Cerrado	42.2	118.6	$9.33x10^{8}$	$2.34x10^{9}$	$2.68x10^{7}$	$6.35x10^{7}$	$2.68x10^{7}$	$6.35x10^{7}$		
**Productive lands in the Forest biome**										
Permanent productive for 30 years +	24.2	62	$2.11x10^{7}$	$5.23x10^{7}$	$1.90x10^{5}$	$5.93x10^{5}$	$1.90x10^{5}$	$5.93x10^{5}$		
Permanent productive for maximum of 30 years	33.1	67	$1.05x10^{8}$	$2.07x10^{8}$	$6.32x10^{5}$	$1.03x10^{6}$	$6.32x10^{5}$	$1.03x10^{6}$		
Permanent productive for maximum of 20 years	48.6	76.5	$2.83x10^{8}$	$4.36x10^{8}$	$3.15x10^{6}$	$4.24x10^{6}$	$3.15x10^{6}$	$4.14x10^{6}$		
Under consolidation (productive for 10 years or less)	48.6	101.9	$2.77x10^{8}$	$5.79x10^{8}$	$2.80x10^{6}$	$5.67x10^{6}$	$2.80x10^{6}$	$5.67x10^{6}$		
Deforestation in 2010	89.7	137.8	$7.41x10^{5}$	$1.14x10^{6}$	$6.46x10^{3}$	$5.87x10^{3}$	$3.23x10^{3}$	$2.94x10^{3}$	$1.07x10^{4}$	$9.69x10^{3}$
**Productive lands in the Cerrado biome**										
Permanent productive for 30 years +	16.2	60.1	$4.87x10^{7}$	$1.65x10^{8}$	$6.03x10^{5}$	$1.94x10^{6}$	$6.03x10^{5}$	$1.94x10^{6}$		
Permanent productive for maximum of 30 years	17.2	59.7	$1.16x10^{8}$	$3.94x10^{8}$	$6.31x10^{5}$	$2.38x10^{6}$	$6.31x10^{5}$	$2.38x10^{6}$		
Permanent productive for maximum of 20 years	21.3	63.6	$1.86x10^{8}$	$5.38x10^{8}$	$1.65x10^{6}$	$3.77x10^{6}$	$1.65x10^{6}$	$3.77x10^{6}$		
Under consolidation (productive for 10 years or less)	21.4	63.5	$6.38x10^{7}$	$1.82x10^{8}$	$3.38x10^{5}$	$1.15x10^{6}$	$3.28x10^{5}$	$1.15x10^{6}$		
**Regrowth**										
Cerrado	23	90.7	$4.08x10^{6}$	$5.37x10^{6}$	$6.02x10^{4}$	$2.12x10^{5}$	$6.02x10^{4}$	$2.12x10^{5}$		
Forest regrowth (less than 20 years)	104.7	141.4	$1.49x10^{8}$	$2.0 x10^{8}$	$2.89x10^{3}$	$6.83x10^{3}$	$4.51x10^{2}$	$1.07x10^{3}$	$8.04x10^{3}$	$1.90x10^{4}$
Forest regrowth (less than 10 years)	79.8	181.3	$3.98x10^{5}$	$9.1 x10^{5}$	$1.12x10^{6}$	$1.54x10^{6}$	$1.75x10^{5}$	$2.41x10^{5}$	$3.12x10^{6}$	$4.30x10^{6}$
Deforestation in 2010 on less than 10 years regrowth	59.9	99.7	$7.41x10^{5}$	$2.5x10^{7}$	$5.26x10^{5}$	$7.25x10^{5}$	$2.63x10^{5}$	$3.63x10^{5}$	$8.68x10^{5}$	$1.20x10^{6}$
**Total**			**$6.56x10^{9}$**	**$1.14x10^{10}$**	**$6.01x10^{7}$**	**$1.17x10^{8}$**	**$4.07x10^{7}$**	**$8.98x10^{7}$**	**$6.04x10^{7}$**	**$9.11x10^{7}$**
